# FMRP Regulates the Nuclear Export of *Adam9* and *Psen1* mRNAs: Secondary Analysis of an *N*^6^-Methyladenosine Dataset

**DOI:** 10.1038/s41598-020-66394-y

**Published:** 2020-07-01

**Authors:** Cara J. Westmark, Bryan Maloney, Reid S. Alisch, Deborah K. Sokol, Debomoy K. Lahiri

**Affiliations:** 1 Department of Neurology, University of Wisconsin-Madison, Madison, WI USA; 2 Department of Psychiatry, Indiana Alzheimer Disease Center, Stark Neuroscience Research Institute, Indiana University School of Medicine, Indianapolis, IN USA; 3 Department of Neurological Surgery, University of Wisconsin-Madison, Madison, WI USA; 4 Department of Neurology, Indiana University School of Medicine, Indianapolis, IN USA; 5 Department of Medical and Molecular Genetics, Indiana University School of Medicine, Indianapolis, IN USA

**Keywords:** Methylation, Alzheimer's disease

## Abstract

Fragile X mental retardation protein (FMRP) binds to and regulates the translation of amyloid-β protein precursor (*App*) mRNA, but the detailed mechanism remains to be determined. Differential methylation of *App* mRNA could underlie FMRP binding, message localization and translation efficiency. We sought to determine the role of FMRP and N^6^-methyladeonsine (m^6^A) on nuclear export of *App* mRNA. We utilized the m^6^A dataset by Hsu and colleagues to identify m^6^A sites in *App* mRNA and to determine if the abundance of message in the cytoplasm relative to the nucleus is altered in *Fmr1* knockout mouse brain cortex. Given that processing of APP to Aβ and soluble APP alpha (sAPPα) contributes to disease phenotypes, we also investigated whether *Fmr1*^*KO*^ associates with nuclear export of the mRNAs for APP protein processing enzymes, including β-site amyloid cleaving enzyme (Bace1), A disintegrin and metalloproteinases (Adams), and presenilins (Psen). *Fmr1*^KO^ did not alter the nuclear/cytoplasmic abundance of *App* mRNA. Of 36 validated FMRP targets, 35 messages contained m^6^A peaks but only *Agap2* mRNA was selectively enriched in *Fmr1*^*KO*^ nucleus. The abundance of the APP processing enzymes *Adam9* and *Psen1* mRNA, which code for a minor alpha-secretase and gamma-secretase, respectively, were selectively enriched in wild type cytoplasm.

## Introduction

Reduced expression of fragile X mental retardation protein (FMRP) results in the neurodevelopmental disorder fragile X syndrome (FXS), which is characterized by intellectual disability, autistic-like behaviors and seizures^1^. FMRP is an mRNA binding protein that binds to hundreds of mRNA ligands with dozens of these targets under study in relation to aberrant synaptic function and/or as drug targets for FXS^2–9^. A mouse model that lacks expression of FMRP has been generated (*Fmr1*^*KO*^ mice) and serves as a surrogate for the study of FMRP function^10^. A major structure-function relationship of FMRP is that this RNA binding protein associates with the coding region sequence of transcripts and functions to stall ribosomal translocation^11^, albeit other functions have been identified including differential transport of methylated mRNA out of the nucleus^9,12^.

Methylation is a reversible modification involving the addition of methyl groups to DNA or RNA. In RNA, *N*^6^-methyladenosine (m^6^A) is the most abundant methylation modification in eukaryotes, accounting for more than 80% of RNA methylation. Fifteen percent of methylation consensus motifs are m^6^A modified with enrichment at the 5’-UTR, near stop codons, in the 3’-UTR, and within long exons at an estimated average level of three m^6^A residues per mRNA^13^. RNA m^6^A modification occurs in the nucleus concurrent with transcription and serves as a chemical imprint that affects mRNA metabolism^14^. Specifically, mRNA m^6^A methylation has the potential to affect RNA folding, splicing, stability, sorting, transport, localization, storage, degradation and translation^14–16^. FMRP is a nucleocytoplasmic shuttling protein that binds mRNAs in the nucleus^17^, and has roles in many of the same aforementioned methylation-based functions. Thus, it is of interest to determine if methylation affects crosstalk between FMRP and its mRNA targets.

Hsu and colleagues recently combined photoactivatable ribonucleoside-enhanced cross-linking and immunoprecipitation (PAR-CLIP) with m^6^A immunoprecipitation (m^6^A-IP) to determine if FMRP binds directly to m^6^A methylation modifications on messenger RNA (mRNA)^18^. They demonstrated that FMRP binds directly to m^6^A sites in mRNAs, FMRP deletion increases nuclear m^6^A-mRNA levels, and the abundance of FMRP mRNA targets in the cytoplasm relative to the nucleus decreases in *Fmr1*^*KO*^ mice^18^. These results strongly suggest that FMRP functions in the nuclear export of m^6^A-modified FMRP-target mRNAs.

The mRNA coding for amyloid-β precursor protein (APP) is an FMRP target. FMRP binds to a guanine-rich sequence in the coding region of both the mouse (*App*) and human (*APP*) variants of *App* mRNA and inhibits protein synthesis^19,20^. APP is the parent protein that is processed by secretases to produce amyloid-β (Aβ), which is the most prevalent protein found in the senile plaques of Alzheimer’s disease, as well soluble APP alpha (sAPPα), which is elevated in autism^21–23^. APP is dysregulated in *Fmr1*^*KO*^ mice through a metabotropic glutamate receptor 5 (mGluR_5_)-dependent pathway, whereby activation of mGluR_5_ rapidly displaces FMRP from the coding region of *App* mRNA and thus increases translation of APP^24^. The detailed mechanism through which FMRP represses translation of APP remains to be determined.

We hypothesize that FMRP regulates localization, and hence protein synthesis of *App* mRNA through an m^6^A-dependent pathway. Furthermore, differential methylation of *App* mRNA, and not variations in FMRP levels or activity, could explain cases of autism spectrum disorder that do not accompany FMRP aberrations. Thus, cross-talk between FMRP and m^6^A-*App* mRNA could have implications for FXS, Alzheimer’s disease, and autism. Here, we utilized the Supplementary Information provided by Hsu and colleagues to identify m^6^A sites in *App* mRNA and to determine if the abundance of message in the cytoplasm relative to the nucleus is altered in *Fmr1* knockout (KO) mouse brain cortex. Given that processing of APP may also contribute to disease-associated differences in the APP metabolites Aβ and sAPPα, we also investigated whether *Fmr1*^*KO*^ associates with nuclear export of the mRNAs for APP processing enzymes, including β-site amyloid cleaving enzyme 1 (Bace1) and A disintegrin and metalloproteinases (Adam) 9, 10, and 17.

## Results

**Figure 1:**
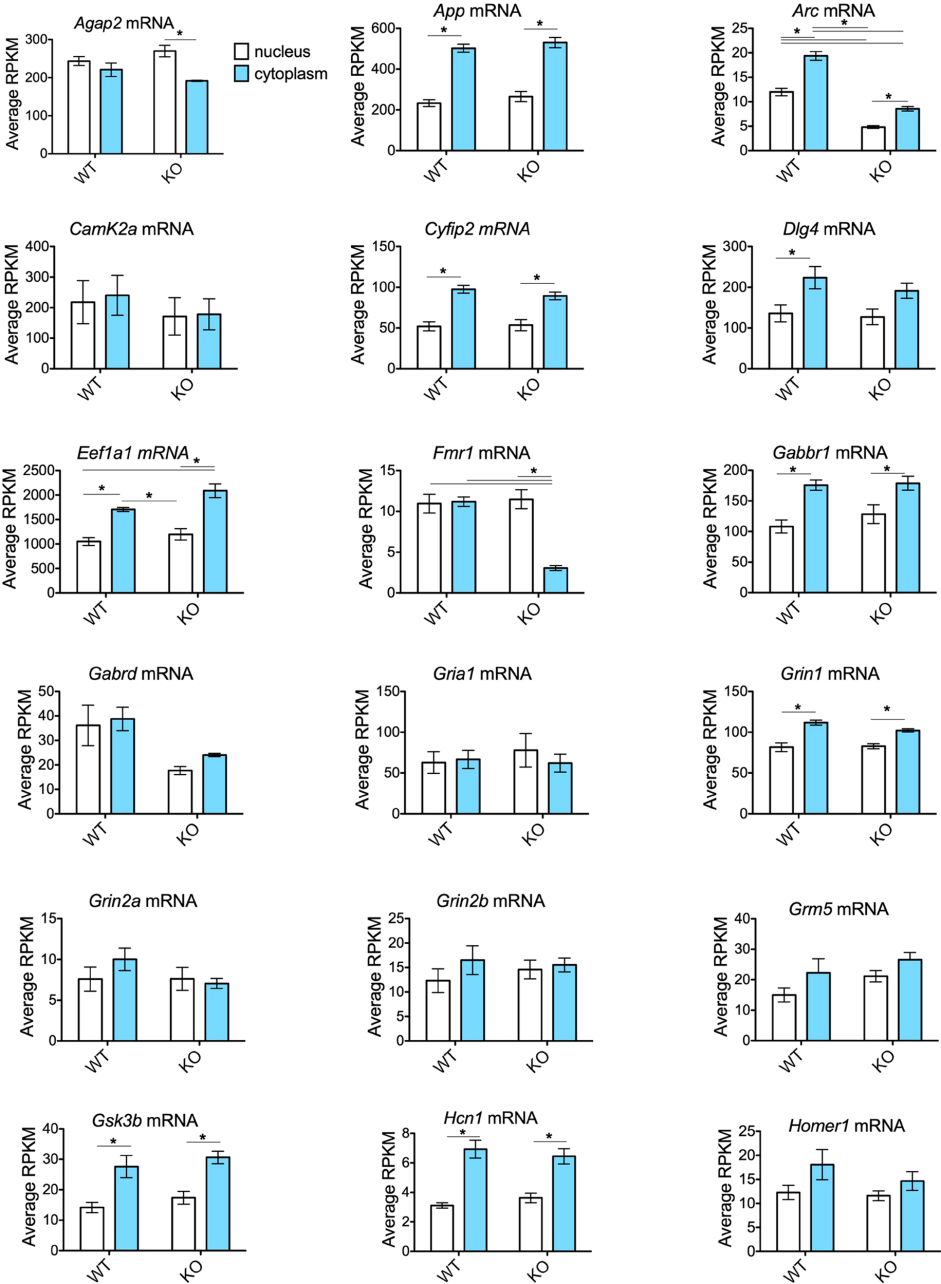
Nuclear and cytoplasmic distribution of FMRP target mRNAs. Using the m^6^A-Seq dataset generated by Hsu and colleagues (Hsu Supplementary Table 5 ^18^), RPKM values were extracted for nuclear and cytoplasmic fractions isolated from cortices of WT and *Fmr1*^*KO*^ mice (postnatal day 11) and the mean expression level was plotted as response variable versus mouse genotype as predictor. Error bars represent standard error of the mean (SEM). Asterisks indicate statistical differences between nuclear and cytoplasmic compartments computed by 2-way ANOVA with *post-hoc* Bonferroni multiple comparison tests (*p* < 0.050). Screened FMRP targets were previously reviewed^9^. Targets are presented in alphabetical order. See Figure 2 for the remaining targets.

The relative abundance of *App* mRNA in the cytoplasm versus the nucleus, based on RNAseq in cortical tissue from wild type (WT) and *Fmr1*^*KO*^ C57BL/6 J mice (postnatal day 11), indicated significantly increased abundance of *App* mRNA in the cytoplasm that did not change in response to *Fmr1* knockdown (Fig. 1, Supplementary Table S1). The reported data were in reads per kilobase per million (RPKM), which normalizes the RNAseq data for both sequencing depth and the length of the gene (Hsu Supplementary Information Table S5^18^).

**Figure 2:**
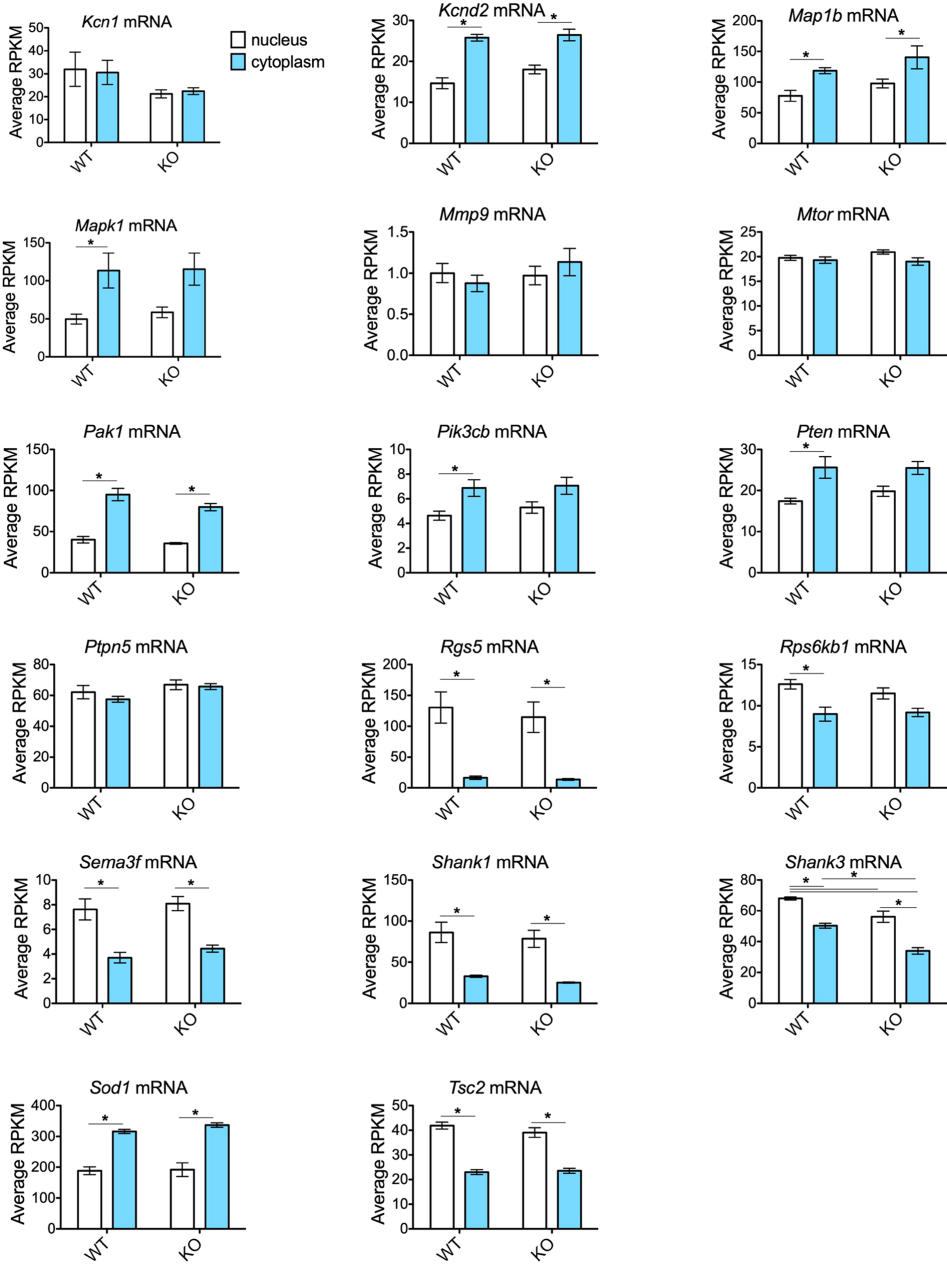
Nuclear and cytoplasmic distribution of FMRP target mRNAs. Using the m^6^A-Seq dataset generated by Hsu and colleagues (Hsu Supplementary Table 5 ^18^), RPKM values were extracted for nuclear and cytoplasmic fractions isolated from cortices of WT and *Fmr1*^*KO*^ mice (postnatal day 11) and the mean expression level was plotted as response variable versus mouse genotype as predictor. Error bars represent standard error of the mean (SEM). Asterisks indicate statistical differences between nuclear and cytoplasmic compartments computed by 2-way ANOVA with *post-hoc* Bonferroni multiple comparison tests (*p* < 0.050). Screened FMRP targets were previously reviewed^9^. Targets are presented in alphabetical order. See Figure  1 for the remaining targets.

Methylation profiling of mouse cortical tissue identified multiple m^6^A sites in *App* mRNA (Table 1). There appears to be two highly reproducible m^6^A sites at 84–211 and 1222–1390, with additional sites at 536–600 and 878–1017. The first m^6^A site encompasses the ATG start codon at position 150, and the other three sites are within the coding region of *App* mRNA (NM_001198823). The 878–1017 methylation site is immediately downstream of a near canonical G-quartet sequence in the coding region of *App* mRNA (position 825–846; X59379)^19^. The average Log2 enrichment was high for two of the four sites including the 84–211 site encompassing the start codon of *App* mRNA and the 1222–1390 site in the coding region (Table 2).

**Table 1:** m6A Profiling of *App* mRNA in Mouse Cortex^a^.

Genotype	Location	Animal	Binding Sites in NM_001198823	Log2 Enrichment
WT	Nucleus	1	84–251 536–750 1220–1414	3.99 1.09 4.12
WT	Nucleus	2	61–247 1222–1435	4.08 3.71
KO	Nucleus	1	61–249 461–600 1220–1413	4.45 0.99 4.13
KO	Nucleus	2	76–233 851–1017 1220–1414	6.69 4.80 3.78
WT	Cytoplasm	1	53–223 1211–1400	5.59 4.37
WT	Cytoplasm	2	46–211 878–1017 1218–1408	5.38 4.40 4.12
KO	Cytoplasm	1	53–233 1210–1390	4.61 4.11
KO	Cytoplasm	2	61–233 1210–1398	4.71 4.55

^a^Data extracted from Hsu Supplementary Table 3 ^18^.

**Table 2:** *App* mRNA Average Log2 Enrichment ± SEM based on Table 1 data.

Site	Location	Instances^a^	Log2 Enrichment^b^	Fold Enrichment
1: 84–211	start site	2/2/2/2	4.94 ± 0.32	24
2: 536–600	coding sequence	1/0/1/0	0.26 ± 0.17	0.07
3: 878–1017	coding sequence	0/1/1/0	1.15 ± 0.75	1.3
4: 1222–1390	coding sequence	2/2/2/2	4.11 ± 0.10	17

^a^Instances are appearances for WT-nucleus/WT-cytoplasm/KO-nucleus/KO-cytoplasm, maximum 2 appearances per group.

^b^Mean values and SEM are calculated as if “missing sites” had a Log2 enrichment of zero.

The majority of validated FMRP targets contained m^6^A sites, but *Fmr1*^*KO*^ did not alter the abundance of these messages in the cytoplasm relative to the nucleus. Using the FMRP target list prepared by Sethna and colleagues^9^, we found that 35 out of 36 known FMRP target mRNAs (all but *Sapap3/4* mRNA) contained m^6^A peaks (Figs. 1 and 2). In comparison, of the 24,661 screened mRNAs in the dataset, 12% did not contain any m^6^A peaks. The 35 m^6^A-containing FMRP target mRNAs can be grouped based on nuclear and cytoplasmic localization. Twelve mRNAs including *App* mRNA had statistically significantly more message in the cytoplasm than the nucleus in both WT and *Fmr1*^*KO*^ cortex. Five mRNAs had statistically significant more message in the nucleus compared to the cytoplasm in both WT and *Fmr1*^*KO*^ cortex. Eleven mRNAs did not differ between cytoplasm and nuclear localization in WT or *Fmr1*^*KO*^ cortex. Four mRNAs (*Dlg4*, *Mapk1*, *Pk3cb*, and *Pten*) exhibited significantly increased cytoplasmic levels selectively in WT. Finally, one mRNA (*Rps*6*kb1*) exhibited significantly increased nuclear levels selectively in WT. *Fmr1* mRNA levels were low in *Fmr1*^*KO*^ cytoplasm. A single message (ArfGAP with GTPase domain; *Agap2*) was significantly enriched in the nucleus of *Fmr1*^*KO*^, suggesting that loss of FMRP reduced nuclear export. Only five mRNAs (*Arc*, *Eef1a1*, *Fmr1*, *Gabr*d, *Shank3*) exhibited genotype-specific differences by 2-way ANOVA (Supplementary Table S1).

**Figure 3:**
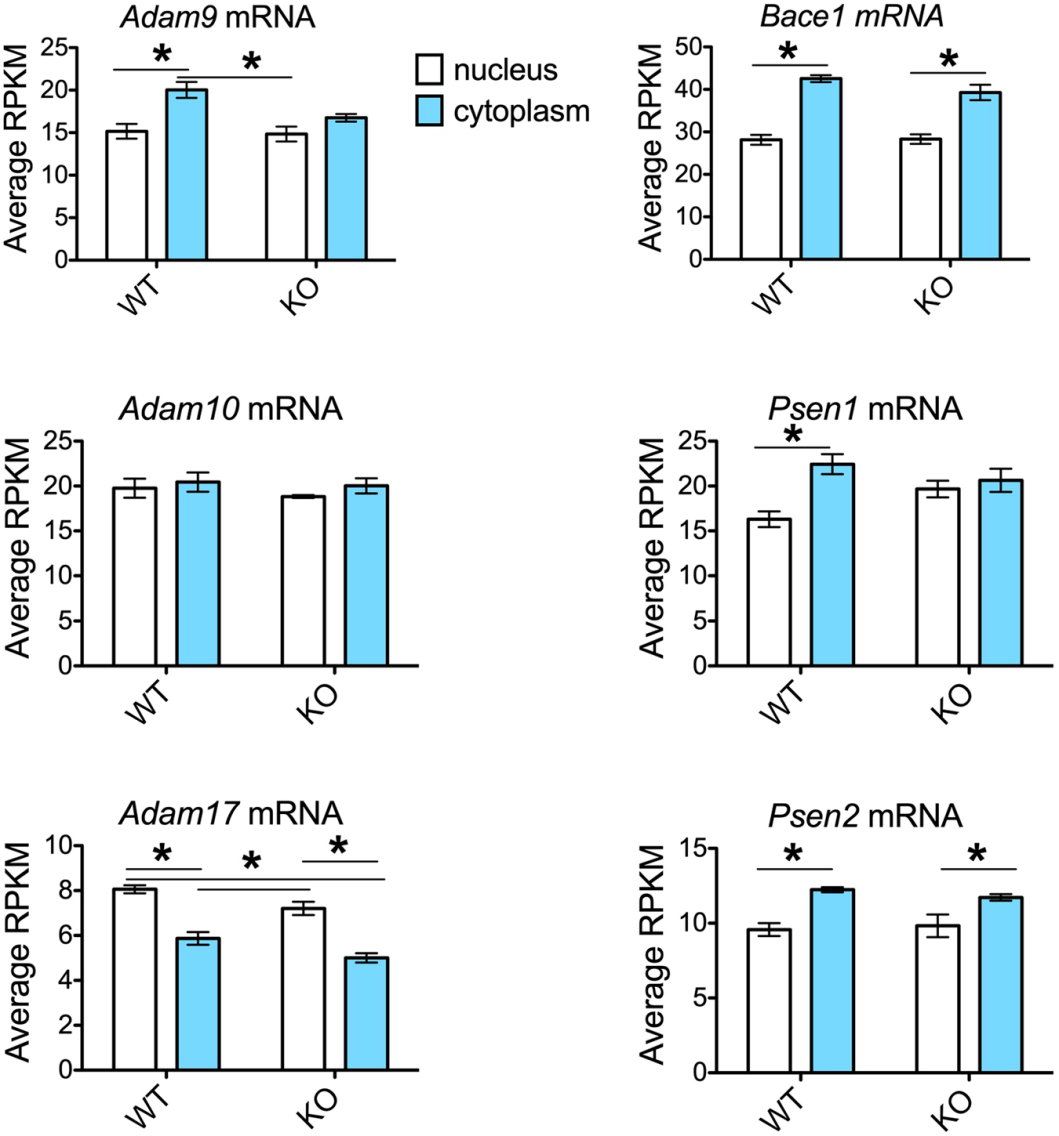
Nuclear and cytoplasmic distribution of mRNAs coding for secretases (*Adam9*, *Adam10*, *Adam17*, and *Bace1*) mRNAs. Using the m^6^A-Seq dataset generated by Hsu and colleagues (Hsu Supplementary Table 5 ^18^), RPKM values were extracted for nuclear and cytoplasmic fractions isolated from cortices of WT and *Fmr1*^*KO*^ mice (postnatal day 11) and the mean expression level was plotted as response variable versus mouse genotype as predictor. Error bars represent standard error of the mean (SEM). Asterisks indicate statistical differences between nuclear and cytoplasmic compartments computed by 2-way ANOVA with *post-hoc* Bonferroni multiple comparison tests (*p* < 0.050).

The mRNAs for APP processing enzymes contained altered nuclear/cytoplasmic abundance as a function of *Fmr1*^*KO*^ status. For *Adam9* and *Psen1*, WT mRNA levels were significantly increased in the cyptoplasm versus nucleus (Fig. 3, Supplementary Table S2). *Fmr1*^KO^ reduced this difference to non-significant levels. *Adam10* mRNA levels did not differ by genotype or location. Levels of *Adam17* mRNA were significantly lower in the cytoplasm compared to the nucleus for both WT and *Fmr1*^KO^ animals. Levels of *Bace1* and *Psen2* mRNAs were significantly higher in cytoplasm than nucleus, but genotype did not exert a significant effect. Given the effects of *Fmr1*^*KO*^ on *Adam9* and *Psen1* mRNA localization, we examined methylation profiling, which identified five m^6^A sites in both *Adam9* (Tables 3 & 4) and *Psen1* mRNAs (Tables 5 & 6). In *Adam9* mRNA, four sites in the coding region were highly reproduced at position 62–184 (crossing start codon), 676–774, 1331–1392, and 2421–2503. The 1331–1392 site was immediately downstream of a GGACU element at nucleotide 1308. In *Psen1* mRNA, three sites were highly reproduced at positions 456–616 (crossing start codon), 1474–1619 (coding sequence), and 2011–2172 (3’UTR). GGACU elements were located at positions 2014, 2025 and 2072 in the 3’-UTR.

**Table 3:** m6A Profiling of *Adam9* mRNA in Mouse Cortex^a^.

Genotype	Location	Animal	Binding Sites in NM_001270996	Log2 Enrichment
WT	Nucleus	1	44–280	3.00
			663–830	1.50
			1293–1459	1.68
			2381–2503	1.76
WT	Nucleus	2	41–197	4.26
			654–789	1.43
			1294–1478	1.88
			2371–2503	1.58
WT	Cytoplasm	1	28–184	6.01
			631–785	1.39
			1236–1392	1.22
			2391–2503	1.10
WT	Cytoplasm	2	45–201	6.01
			618–774	2.12
			1295–1456	1.68
			1819–2006	1.40
			2389–2503	1.80
KO	Nucleus	1	62–216	3.72
			634–789	2.16
			1294–1461	1.98
			2401–2503	1.49
KO	Nucleus	2	43–199	4.91
			1291–1405	1.51
			2391–2503	1.70
KO	Cytoplasm	1	51–206	5.51
			676–831	1.81
			1311–1457	1.07
			2421–2520	1.32
KO	Cytoplasm	2	37–193	5.03
			623–782	1.83
			1331–1474	1.06

^a^Data extracted from Hsu Supplementary Table 3 ^18^.

**Table 4:** *Adam9* mRNA Mean Log 2 Enrichment ± SEM based on Table 3 data.

Site	Location	Instances^a^	Log2 Enrichment^b^	Fold Enrichment
1: 62–184	start site	2/2/2/2	4.81 ± 0.38	23
2: 676–774	coding	2/2/1/2	1.53 ± 0.24	2.3
3: 1331–1392	coding	2/2/2/2	1.51 ± 0.13	2.3
4: 1819–2006	coding	0/1/0/0	0.18 ± 0.18	0.03
5: 2421–2503	coding	2/2/2/1	1.34 ± 0.21	1.8

^a^Instances are appearances for WT-nucleus/WT-cytoplasm/KO-nucleus/KO-cytoplasm, maximum 2 appearances per group.

^b^Mean values and SEM are calculated as if “missing” sites had a Log2 enrichment of zero.

**Table 5:** m6A Profiling of *Psen1* mRNA in Mouse Cortex^a^.

Genotype	Location	Animal	Binding Sites in NM_008943^b^	Log2 Enrichment
WT	Nucleus	1	297–434	3.82
			435–638	2.76
			1465–1627	1.44
			2010–2177	5.57
WT	Nucleus	2	423–638	2.74
			639–758	1.38
			1465–1619	2.04
			2010–2178	5.91
WT	Cytoplasm	1	347–446	3.28
			447–630	2.32
			1474–1629	1.80
			2010–2178	5.46
WT	Cytoplasm	2	337–436	3.40
			447–638	2.36
			1473–1627	1.91
			2006–2175	5.25
KO	Nucleus	1	455–638	3.03
			1444–1626	2.17
			2010–2177	5.65
KO	Nucleus	2	369–621	2.38
			1444–1619	2.43
			1998–2172	6.60
KO	Cytoplasm	1	368–616	2.70
			1472–1628	1.78
			2011–2177	5.84
KO	Cytoplasm	2	337–436	3.39
			456–638	2.54
			639–759	1.43
			1452–1619	1.91
			2009–2177	5.17

^a^Data extracted from Hsu Supplementary Table 3 ^18^.

^b^This RefSeq was removed from NCBI due to insufficient support for the transcript.

**Table 6:** *Psen1* mRNA Mean Log 2 Enrichment ± SEM based on Table 5 data.

Site	Location	Instances^a^	Log2 Enrichment^b^	Fold Enrichment
1: 347–434	5’-UTR	1/2/0/1	1.74 ± 0.66	3.0
2: 456–616	start site	2/2/2/2	2.60 ± 0.087	6.8
3. 639–758	coding	1/0/0/1	0.35 ± 0.23	0.12
4. 1474–1619	coding	2/2/2/2	1.94 ± 0.10	3.8
5. 2011–2172	3’-UTR	2/2/2/2	5.68 ± 0.16	32

^a^Instances are appearances for WT-nucleus/WT-cytoplasm/KO-nucleus/KO-cytoplasm, maximum 2 appearances per group.

^b^Mean values and SEM are calculated as if “missing” sites had a Log2 enrichment of zero.

UTR = untranslated region.

## Discussion

Methylation at m^6^A is the most abundant post-transcriptional mRNA modification in polyadenylated mRNAs and long non-coding RNAs in higher eukaryotes^25^. Recent findings indicate that FMRP target mRNAs contain an increased number of m^6^A peaks, mostly enriched in the coding regions of genes^26^, and that FMRP functions as an m^6^A reader protein that modulates neuron differentiation and mRNA stability through m^6^A-dependent mRNA mechanisms^12,26–29^. Out of 842 FMRP mRNA targets identified by Darnell and colleagues^11^, 95% had m^6^A modifications in mouse brain cerebellum and 96% in cortex^26^. *App* mRNA is a validated FMRP target^19^.

*App* gene expression is negatively regulated by cytosine methylation^30–32^, but little is known regarding methylation-dependent regulation of *App* mRNA other than that small nuclear ribonucleoprotein (SNRP) splicing factors regulate alternative splicing through a methylation-dependent mechanism^17,33^. To our knowledge, nuclear-cytoplasmic transport of *App* mRNA has not been reported^34^. Hsu and colleagues performed m^6^A-Seq in cytoplasmic and nuclear samples from P11 cortical tissue isolated from WT and *Fmr1*^*KO*^ C57BL/6 J mice, and provided the normalized dataset as Supplementary Information to their manuscript^18^. Based on their global analysis of the dataset, they propose that FMRP is an m^6^A reader protein that binds directly to m^6^A sites in mRNA and functions in the export of those messages to the cytoplasm. This is an important phenomenon that could underlie FXS pathogenesis; thus, we wanted to determine if cross-talk between FMRP and m^6^A methylation affects the nuclear export of *App* mRNA.

We found that *App* mRNA contains four m^6^A sites and is more abundant in the cytoplasm relative to the nucleus. *Fmr1*^KO^ did not alter the abundance of *App* mRNA in the cytoplasm or the nucleus suggesting that crosstalk between FMRP and m^6^A sites does not regulate nuclear-cytoplasmic transport of this message. It is not surprising that *App* mRNA levels were similar between WT and *Fmr1*^*KO*^ samples as we previously demonstrated that *App* mRNA is a stable message and altered protein levels are not dependent on message decay^19^.

It is of interest that there is high enrichment of m^6^A in *App* mRNA in the region encompassing the start codon but not at the near canonical G-quartet region. RNAs that contain m^6^A can bind eukaryotic initiation factor 3 (eIF3) without having a 5’-cap. This may facilitate additional cap-independent mRNA translation during cell stress^35^. In addition, the *App* m^6^A region that crosses the start codon also includes a nexus with an overlapping interleukin-1 acute box, an iron response element and a target for microRNA-346, all of which may participate in neuronal iron (Fe) homeostasis^36^. The guanine-rich sequence in the coding region of *App* mRNA functions as a binding site for FMRP and heterogeneous nuclear ribonucleoprotein C (hnRNP C), which compete for binding and inversely regulate APP protein synthesis^20^. FMRP represses translation by recruiting *App* mRNA to processing bodies whereas hnRNP C promotes translation by displacing FMRP^20^. It remains to be determined if m^6^A modification regulates *App* mRNA nuclear export through hnRNP C or other RBP, which may vary as a function of development and disease. PAR-CLIP previously identified three FMRP binding sites in *APP* mRNA (Ascano Supplementary Fig. 7: site 1: 888–948 in the coding region, site 2 in the coding region: 2169–2228, site 3 in the 3’-UTR: 3337–3396)^37^. Site 1 overlaps with the guanine-rich site previously identified in mouse. The other two sites were not identified as m^6^A peaks in the Hsu dataset^18^. Overall, the findings indicate that FMRP does not regulate nuclear-cytoplasmic transport of *App* mRNA through an m^6^A-dependent pathway.

We further asked if the nuclear/cytoplasmic transport of other known FMRP targets or APP secretases were regulated by FMRP/m^6^A crosstalk. Of 36 validated FMRP targets^9^, 35 messages contained m^6^A peaks. Several FMRP target mRNAs (*Dlg4*, *Mapk1*, *Pik3cb*, *Pten* and *Rps6kb1*) exhibited significantly altered nuclear/cytoplasmic distribution in WT samples, but there were trends for the same phenomenon in the *Fmr1*^*KO*^, suggesting that FMRP/m^6^A crosstalk does not play a prominent role in nuclear transport of these messages. Only *Agap2* mRNA was selectively enriched in *Fmr1*^*KO*^ nucleus suggesting that loss of FMRP reduced its nuclear export. *Agap2* mRNA codes for phosphoinositide-3 kinase enhancer (PIKE) protein, which is an important regulator of group 1 mGluR-dependent phosphoinositide-3 kinase (PI3K) activity^38,39^. The gene for *Agap2* is highly enriched in key pathways involved in amyloid-beta formation, the regulation of cardiocyte differentiation, and in actin cytoskeleton reorganization^40^. The *Agap2* promoter is hypermethylated in Alzheimer’s disease^41^. *Agap2* mRNA was not included in the Edupuganti pulsed-SILAC translation dataset^29^, suggesting that FMRP regulates nuclear export but not protein synthesis. Of the 36 validated FMRP mRNA targets reviewed by Sethna and colleagues^9^, only 5 are present in the Edupuganti dataset (*EEF1A1*, *FMR1*, *GSK3B*, *MAPK1*, *SOD1*).

**Figure 4:**
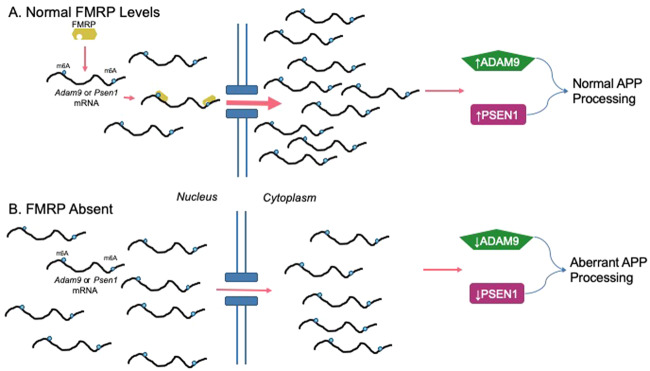
Potential function of FMRP in regulating the transport of *Adam9* and *Psen1* mRNA into the cytoplasm. (**A**) Under normal conditions, FMRP recognizes m^6^A sites on multiple mRNAs, including *Adam9* and *Psen1*, and interacts with the mRNA transport machinery (not shown). Transported mRNAs are available for protein synthesis resulting in normal levels of ADAM9 and PSEN1 protein and normal APP processing. (**B**) The absence of FMRP leads to reduced transport of m^6^A-marked mRNAs, potentially reducing levels of ADAM9 and PSEN1 proteins. While ADAM10 activity may compensate, disruption of the gamma-secretase complex may result in subtle cell dysfunction.

*Adam9* mRNA, which encodes for a minor α-secretase, as well as *Psen1* mRNA, which codes for gamma secretase, were selectively reduced in the nucleus of WT samples but not *Fmr1* knockouts, suggesting that FMRP may play a role in cytoplasmic transport of these secretase coding mRNAs (Fig. 4). This finding is unexpected in light of western blot data showing equal ADAM9 protein levels between WT and *Fmr1*^KO^ and lack of FMRP/*Adam9* mRNA co-immunoprecipitation^42^ even though *Adam9* mRNA possess a near canonical G-quartet (DWGGN_0–2_DWGGN_0–1_DWGGN_0–1_DWGG)^7^ at position 3756 in the 3’-UTR (TAGG_CT_GGAG_A_AAGG_AAGG) (NM_001270996). Deletion of ADAM9 does not appreciably alter levels of α-secretase processing of APP^43^, but this may be due to compensatory upregulation of ADAM10^44^. An in-depth investigation of ADAM9 protein or mRNA levels in human subjects with APP-related disorders, such as Alzheimer’s disease and autism spectrum disorder, has yet to be performed. It may be possible that ADAM9 disruption functions in some but not all APP-related disorders.

Overall, the main findings of this study were that FMRP/m^6^A crosstalk does not mediate the nuclear export of *App* mRNA nor export of the majority of other validated FMRP target mRNAs, but does affect the nuclear export of mRNAs for two APP secretases, *Adam9* and *Psen1*. The function of m^6^A sites in *Adam9* and *Psen1* messages remains to be determined. Specifically, mRNA methylation has the potential to affect RNA folding, splicing, stability, sorting, transport, localization, storage, degradation and/or translation^14–16^. Disruption of ADAM9 function could play a role in some but not all APP-related disorders. Further investigation of ADAM9, AGAP2 and PSEN1 levels in human subjects with APP-related disorders could help in understanding Alzheimer’s disease and autism spectrum disorders. It also remains to be determined how the binding and activity of other RBP are affected by m^6^A methylation and if m^6^A methylation is altered as a function of development and environment. The limitation of this study is the dataset is dependent on one time point, which precludes analysis as a function of development and disease severity. The strengths of the study are the large dataset, nuclear/cytoplasmic distribution data in quadruplicate, and utilization of the most widely used FXS model.

## Methods

Dataset: We utilized the m^6^A dataset generated by Hsu and colleagues, which is available online at http://www.jbc.org/content/294/52/19889.long, to extract data regarding m^6^A modifications to *App*, *Adam9* and *Psen1* mRNAs (Hsu Supplementary Table 3^18^) as well as FMRP target mRNA nuclear/cytoplasmic distributions (Hsu Supplementary Table 5 ^18^). The Hsu dataset was generated by performing RNA isolation and m^6^A-Seq on nuclear and cytoplasmic fractions isolated from cortices of wild type (WT) and *Fmr1*^*KO*^ mice in the C57BL/6 J background (postnatal day 11). m6A-Seq data were available for 23,869 mRNAs and nuclear/cytoplasmic distribution data were available for 24,661 mRNAs. m6A-Seq was performed in duplicates and nuclear/cytoplasmic distribution in quadruplicate.

Analyses: Data were analyzed in accordance with STROBE guidelines (https://strobe-statement.org/index.php?id=available-checklists). Means, standard deviations from the mean (SEM), and 2-way ANOVA with *post-hoc* Bonferroni multiple comparison tests were computed to describe the results. Statistical significance was defined as *p* < 0.050.

## Supplementary information

Supplemental information.

## Data Availability

All materials and data associated with the manuscript are or will be made available to readers by contacting the corresponding author.
