# Supplementary material for: FMRP Regulates the Nuclear Export of Adam9 and Psen1 mRNAs: Secondary Analysis of an N6-Methyladenosine Dataset
Source: Sci Rep. 2020 Jul 1;10:10781. doi: 10.1038/s41598-020-66394-y (PMC7329887; doi:10.1038/s41598-020-66394-y)
Supplement: Supplementary file 1 — Supplemental information. [file 41598_2020_66394_MOESM1_ESM.pdf]

Supplementary Materials

FMRP Regulates the Nuclear Export of *Adam9* and *Psen1* mRNAs: Secondary Analysis of an *N*[6]-Methyladenosine Dataset

Cara J. Westmark[a*], Bryan Maloney[b], Reid S. Alisch[c], Deborah K. Sokol[d],

and Debomoy K. Lahiri[be]

**Supplementary Table S1: 2-Way ANOVA Stats for FMRP Targets**				
**Target**	**Effect**	**DF**	**F**	***p***
Agap2	Interaction	1	4.527	0.055
	Localization	1	14.78	0.0023
	Genotype	1	0.01328	0.91
App	Interaction	1	0.008719	0.93
	Localization	1	148.8	<0.0001
	Genotype	1	1.886	0.19
Arc	Interaction	1	8.075	0.015
	Localization	1	75.53	<0.0001
	Genotype	1	197.3	<0.0001
CamK2a	Interaction	1	0.01546	0.90
	Localization	1	0.05602	0.82
	Genotype	1	0.7592	0.40
Cyfip2	Interaction	1	0.7861	0.39
	Localization	1	54.76	<0.0001
	Genotype	1	0.3471	0.57
Dlg4	Interaction	1	0.2980	0.60
	Localization	1	12.14	0.0045
	Genotype	1	0.8764	0.37
Eef1a1	Interaction	1	1.359	0.27
	Localization	1	58.71	<0.0001
	Genotype	1	6.792	0.023
Fmr1	Interaction	1	24.47	0.0003
	Localization	1	21.90	0.0005
	Genotype	1	18.82	0.0010
Gabbr1	Interaction	1	0.5333	0.48
	Localization	1	25.60	0.0003
	Genotype	1	0.9911	0.3391
Gabrd	Interaction	1	0.1468	0.71
	Localization	1	0.8574	0.37
	Genotype	1	11.57	0.0053
Gria1	Interaction	1	0.4578	0.51
	Localization	1	0.1671	0.69
	Genotype	1	0.1287	0.73

Grin1	Interaction	1	2.175	0.17
	Localization	1	47.05	<0.0001
	Genotype	1	1.346	0.27
Grin2a	Interaction	1	1.379	0.26
	Localization	1	0.5487	0.47
	Genotype	1	1.338	0.27
Grin2b	Interaction	1	0.5312	0.48
	Localization	1	1.310	0.27
	Genotype	1	0.08171	0.78
Grm5	Interaction	1	0.09686	0.76
	Localization	1	4.546	0.054
	Genotype	1	3.064	0.11
Gsk3b	Interaction	1	0.001853	0.97
	Localization	1	28.85	0.0002
	Genotype	1	1.554	0.24
Hcn1	Interaction	1	1.288	0.28
	Localization	1	56.68	<0.0001
	Genotype	1	0.0009982	0.98
Homer1	Interaction	1	0.4524	0.51
	Localization	1	4.691	0.051
	Genotype	1	1.004	0.34
Kcnc1	Interaction	1	0.07261	0.79
	Localization	1	0.0004405	0.98
	Genotype	1	4.021	0.068
Kcnd2	Interaction	1	1.367	0.27
	Localization	1	70.26	<0.0001
	Genotype	1	3.031	0.11
Map1b	Interaction	1	0.005609	0.94
	Localization	1	13.94	0.0029
	Genotype	1	3.465	0.0873
Mapk1	Interaction	1	0.04553	0.83
	Localization	1	13.68	0.0030
	Genotype	1	0.1054	0.75
Mmp9	Interaction	1	1.303	0.28
	Localization	1	0.02481	0.88
	Genotype	1	0.8267	0.38
Mtor	Interaction	1	1.528	0.24
	Localization	1	4.069	0.067
	Genotype	1	0.5940	0.46
Pak1	Interaction	1	1.271	0.28
	Localization	1	106.7	<0.0001
	Genotype	1	4.245	0.062
Pik3cb	Interaction	1	0.1793	0.68
	Localization	1	12.52	0.0041
	Genotype	1	0.5520	0.47
Pten	Interaction	1	0.5592	0.47
	Localization	1	17.24	0.0013
	Genotype	1	0.4449	0.52

Ptpn5	Interaction	1	0.3189	0.58
	Localization	1	0.9345	0.35
	Genotype	1	4.692	0.051
Rgs5	Interaction	1	0.1314	0.72
	Localization	1	36.71	<0.0001
	Genotype	1	0.2701	0.61
Rps6kb1	Interaction	1	0.9615	0.35
	Localization	1	19.78	0.0008
	Genotype	1	0.4755	0.50
Sema3f	Interaction	1	0.05194	0.82
	Localization	1	42.80	<0.0001
	Genotype	1	1.082	0.32
Shank1	Interaction	1	8.134e-005	0.99
	Localization	1	42.85	<0.0001
	Genotype	1	0.8865	0.37
Shank3	Interaction	1	0.9346	0.35
	Localization	1	76.19	<0.0001
	Genotype	1	38.57	<0.0001
Sod1	Interaction	1	0.3831	0.55
	Localization	1	100.9	<0.0001
	Genotype	1	0.7841	0.39
Tsc2	Interaction	1	1.420	0.26
	Localization	1	152.1	<0.0001
	Genotype	1	0.6660	0.43

**Supplementary Table S2: 2-Way ANOVA Stats for APP Secretase Targets**				
**Target**	**Effect**	**DF**	***F***	***p***
Adam9	Interaction	1	3.405	0.090
	Localization	1	17.76	0.0012
	Genotype	1	5.010	0.045
Adam10	Interaction	1	0.08575	0.77
	Localization	1	1.165	0.30
	Genotype	1	0.5946	0.46
Adam17	Interaction	1	0.001462	0.970
	Localization	1	79.79	<0.0001
	Genotype	1	12.30	0.0043
Bace1	Interaction	1	1.795	0.21
	Localization	1	97.56	<0.0001
	Genotype	1	1.465	0.25
Psen1	Interaction	1	5.827	0.033
	Localization	1	11.01	0.0061
	Genotype	1	0.5349	0.48
Psen2	Interaction	1	0.7181	0.41
	Localization	1	24.97	0.0003
	Genotype	1	0.08379	0.78
